# Application of Chelex-100 and SPR-IDA Resin in Combination with the Optimized Beam Deflection Spectrometry for High-Sensitivity Determination of Iron Species in Sediment Porewater

**DOI:** 10.3390/s25123643

**Published:** 2025-06-10

**Authors:** Hanna Budasheva, Mohanachandran Nair Sindhu Swapna, Arne Bratkič, Dorota Korte

**Affiliations:** 1 Laboratory for Environmental and Life Sciences, University of Nova Gorica, Vipavska 12, 5000 Nova Gorica, Sloveniaswapna.nair@ung.si (M.N.S.S.); 2 National Institute of Biology–Marine Biology Station, Fornače 41, 6330 Piran, Slovenia; 3 Department of Chemistry, Physics, Environmental and Soil Sciences, University of Lleida, Av. Rovira Roure 191, 25198 Lleida, Spain

**Keywords:** iron species, sediment porewater, diffusive gradients in thin film, resins, photothermal, beam deflection spectrometry

## Abstract

In this work, photothermal beam deflection spectrometry (BDS), combined with a passive sampling technique of diffusive gradients in thin film (DGT), is optimized to improve the method’s sensitivity. The limit of detection (LOD) is then reduced by a factor of 2 (to the value of 20 nM). The functionality of the technique is compared for Chelex-100 (Ch-100) and suspended particulate reagent–iminodiacetate resin (SPR-IDA), used as binding resins in passive samplers. The absorption capacity of SPR-IDA resin is found to be less than 1 μM and far below that one of Chelex-100 resin (around 6 μM). The BDS technique is applied for determination of iron redox species concentration in sediment porewater. It is found that Fe in sediment porewater occurs both in Fe^2+^ (0.073 μM) and Fe^3+^ (0.095 μM) forms. The validation of the presented method reveals that the BDS technique ensures good repeatability, reproducibility, and reliability.

## 1. Introduction

Iron (Fe) in aquatic environments occurs in two oxidation states: Fe^2+^ or Fe^3+^ [1]. They are stable thermodynamically under both anoxic (without oxygen) and oxic (oxygen-containing) conditions [2,3,4]. These redox forms may be transferred from one to another in a biotically or abiotically way. These transformations occur at oxic/anoxic boundaries that include the sediment–water interface (SWI) and are factors that determine the circulation of Fe in water. In case of oxic waters, Fe^3+^ reaches a stable oxidation state that, at neutral pH, forms insoluble hydroxides and oxides [5,6,7]. When waters are in the anoxic state, Fe^2+^ species take the stable form of dissolved ions. There is an exception in the case of a water environment with high levels of carbonate, sulfide, and orthophosphate, in which Fe^2+^ forms insoluble salts [8,9]. The process of Fe^3+^ reduction can occur also in oxygenated surface lake freshwaters at high pH [10,11]. The biotic part of the biogeochemical Fe cycle involves a variety of microorganisms [12]. Some of them need Fe^3+^ to work as an electron acceptor in the oxidation process of organic carbon or hydrogen. Another basis of implementation is Fe^2+^ an electron donor that is involved in the reduction process of O_2_ or nitrate. Others use Fe^2+^ as an electron donor for performing photosynthesis where Fe^2+^ oxidation is coupled to CO_2_ reduction in the presence of light as the source of energy [13]. Furthermore, iron binds inorganic dissolved phosphate, which is an essential nutrient in the process of primary production. The complex interaction between Fe and P occurs at SWI and is found to often be redox-controlled. As a result, sediments become significant sinks of phosphate, which causes the complexes with iron species to further influence the process of eutrophication and accumulation of organic matter in the sediment over time [14,15]. Thus, the determination of the amount and distribution of Fe species in aqueous environments is an important factor that explains mechanisms of biogeochemical processes occurring in water environments [16,17].

Investigation of fractions that are labile, dissolved, and accessible to biota (bioavailable) is often limited because of their extremely low concentrations in environmental samples (often less than 1 μM) [18]. A solution to this problem is provided by the diffusive gradients in thin films technique (DGT). It has been found to be suitable for in situ labile fraction sampling and analysis [19,20,21] when combined with a proper detection method, such as atomic absorption spectrometry (AAS) [22,23], electrothermal atomic absorption spectrometry (ETAAS) [24,25], inductively coupled plasma–mass spectrometry (ICP-MS) [26,27], ion chromatography (IC) [28,29], high-performance liquid chromatography with plasma-based optical emission detectors (HPLC–MIP OES) [30], atomic emission spectrometry (AES) [31], total reflection X-ray fluorescence (TXRF) [32,33], proton-induced X-ray emission (PIXE) [34,35], stripping voltammetry (SV) [36,37], chemiluminescence (CL) [38,39], capillary electrophoresis (CE) [40,41], or UV–vis spectrophotometry (UV–vis SPEC) [42]. Unfortunately, all these techniques are applied only for total iron detection but are not suitable for characterization of redox speciation [43]. Also, they provide rather high detection limits (LODs) that exceed the value of 0.1 µM, which is often insufficient for the analysis of environmental samples [44]. A solution of this problem is found in the pre-concentration of samples and/or applying separation techniques for species differentiation. Such solutions require the use of expensive techniques that involve complicated analytical procedures and are sensitive to both spectral and non-spectral interferences. Moreover, there is not only a risk of contamination during the multi-step sample preparation process [45,46] but also a lack of reliable information regarding the content of Fe species [47]. Some of these drawbacks can be avoided using electrochemical methods (EM) that are of relatively low cost and offer the possibility of their miniaturization and automatization for in situ measurements [48,49,50]. EMs applications for Fe content determination are either based on direct Fe^3+^ reduction to Fe^2+^ on the working electrode or on adsorptive cathodic stripping voltammetry, which requires the addition of a ligand to bind Fe and make a complex [51]. EMs provide LODs as low as 0.03 nM [52], but their use is limited in complex media since the measured signal is then impacted by the presence of other species [47].

Promising alternatives are techniques based on photothermal effect (PT). Among them, photothermal beam deflection spectrometry (BDS) is of high interest. It is nowadays widely used to measure the low optical absorption and determine the thermal and related characteristics of different samples, especially those for which transition techniques (e.g., thermal lens spectrometry—TLS) cannot be applied [53,54]. The principle of BDS is related to the photo-induced changes in the thermal state of the examined sample that absorbs the light energy of excitation radiation (EB). The absorbed energy is further realized as heat-inducing temperature oscillations (TOs) in both the sample and in the fluid layer over its surface. This leads to material’s (the sample and the fluid over it) refractive index changes [55,56] that are sensed by a probe laser beam (PB). The PB skims the sample’s surface perpendicular to the EB while propagating through the area of TOs induced in the fluid above the sample’s surface (transverse BDS). Unfortunately, in the case of low sample absorption of the EB and short interaction length of the PB with TOs, the configuration of the BDS experimental setup must be optimized to provide high-sensitivity measurements for a desired application [57,58,59].

The BDS has been already successfully coupled to the DGT sampling technique, providing a two-dimensional distribution of Fe labile redox species in sediments [60] and introducing a baseline for improving the method sensitivity and ease of operation. Thus, the goal of this work is to develop an optimized DGT-BDS technique for non-destructive Fe species characterization and present its effectiveness in applicability to the determination of iron species in river sediments. The optimization of the BDS experimental setup is based on constructing a cavity enabling The PB’s multiple reflections within the area of TOs, which results in the signal enhancement and thus the measurement sensitivity. The details of the BDS-DGT technique optimization are discussed together with presentation of the method validation. Also, an application of the determination procedure is presented.

## 2. Materials and Methods

### 2.1. Chemicals and Solutions

Double-deionized water (ddH_2_O) (18 MΩ·M, NANOPURE) was used to prepare all the solutions. The used reagents were

- 5.9 mL of pure HCl (32%, Sigma-Aldrich, St. Louis, MO, USA), which was diluted to 10 mL in ddH_2_O to obtain 6 M HCl;
- 4.9 mL of 32% HCl, which was diluted to 500 mL in ddH_2_O to prepare 0.1 M HCl;
- 0.6 mL of CH_3_COOH (99.8% purity, Merck, Darmstadt, Germany), which was diluted to 100 mL in ddH_2_O to get 0.1 M CH_3_COOH solution (AA);
- 7.7 g of CH_3_COONa (98.5% purity, Fluka Chemika, Seelze, Germany), which was added to 1 L in ddH_2_O and adjusted with pure CH_3_COOH (99.8%, Merck, New York, NY, USA) to obtain 0.1 M acetic buffer with pH = 5.6;
- 0.132 g of C_6_H_8_O_6_ (99.7% purity, Fischer Scientific, Loughborough, UK), which was dissolved in 250 mL of AA to prepare 0.003 M L-ascorbic acid (C_6_H_8_O_6_) solution (LAA);
- 132 mg of C_6_H_8_O_6_ (99.7% purity, Fischer Scientific, UK), which was dissolved to 250 mL in acetic buffer to get 3 mM LAA;
- 2.7 g of anhydrous 1.10-phenanthroline (99% purity, Merck, Darmstadt, Germany), which was mixed with 5 mL of 6 M HCl and further diluted to 500 mL in ddH_2_O to obtain 30 mM *o*-phen solution;
- 695 mg of ferrous (assay of Fe 86–90%) FeSO_4_·7H_2_O reagent (Merck, Germany), which was dissolved to 250 mL in 0.1 M HCl to prepare 10 mM Fe^2+^ stock solution;
- Working standard solutions of Fe^2+^ in a concentration range from 0.1 to 1 μM, which were made by diluting a proper amount of Fe^2+^ stock solution in ddH_2_O;
- 678 mg of crist. p.a. Fe^3+^ FeCl_3_·6H_2_O) reagent (Riedel de Haen, Seelze, Germany), which was dissolved in 250 mL of 0.1 M HCl solutions to prepare 10 mM Fe^3+^ stock solution;
- Working standard solutions of Fe^3+^ in a concentration range of 0.1–1 μM, which were made by diluting a proper amount of Fe^3+^ stock solution in ddH_2_O.

The reagents were used without additional purification.

### 2.2. SPR-IDA and Ch-100 Resins Preparation

The suspended particulate reagent-iminodiacetate (SPR-IDA) resin was prepared by mixing the commercial binding reagent with a gel solution according to the procedure described in the literature [61,62]. The Chelex-100 resin is made of styrene divinylbenzene copolymers with paired iminodiacetate ions to be chelators for binding metal ions, as can be found in refs. [63,64,65].

### 2.3. Diffusive Gradients in Thin Films (DGT) Coupled to Beam Deflection Spectrometry (BDS)

**Figure 1:**
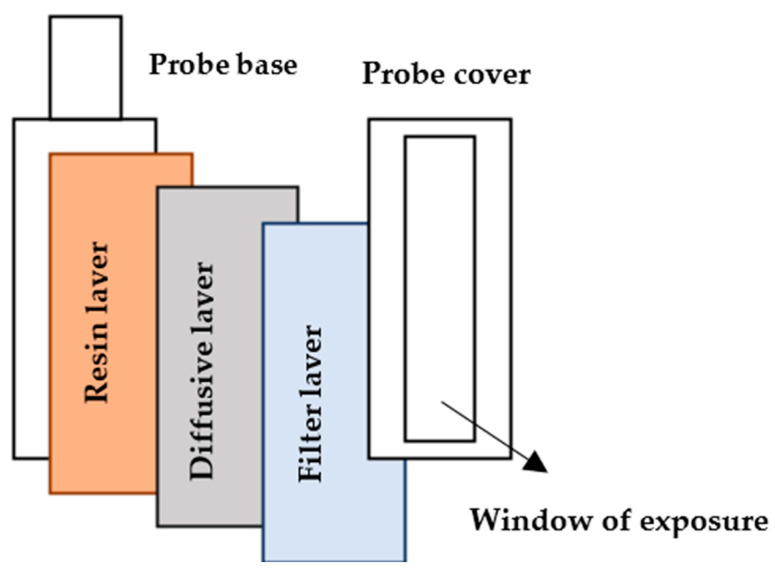
The assembled DGT probe and its components for sediment analysis.

The BDS in combination with the DGT technique is applied for performing the analysis of Fe species concentration and distribution in the sediment porewater [43,44]. The DGT as a dynamic sampling technique is based on the in situ binding of dissolved metal species in an aqueous environment. The DGT probe (Figure 1) [18] contains a diffusive layer (polyacrylamide gel), followed by a protective membrane and a resin bounding gel. After its deployment, the analytes of interest are taken up by the resin bounding gel. The probe is exposed to the water medium for a certain period in which the mass of solute is accumulated in the binding layer of the probe. If the thickness ∆*d* of species diffusion depth is known (the thickness of the diffusive layer that includes the protective membrane), its averaged concentration in an aqueous environment is found as [66]

(1) $$C_{DGT}=M_{\alpha }∆d{\left(DAt\right)}^{-1},$$

where *D* denotes the diffusive coefficient of the analyte in the diffusive layer, *t* defines the deployment time (sampling time), and *M_α_* and *A* are the mass of the accumulated analyte during the sampling time and the resin’s surface area (sampling window area), respectively. The time-averaged fluxes passing through the whole sampler can then be calculated as

(2) $$f_{DGT}=M_{\alpha }{\left(At\right)}^{-1}$$

### 2.4. Sampling

The DGT sediment probes (Figure 1) with either Ch-100 or SPR-IDA (Teledyne CETAC^®^, Omaha, NE, USA) resins as a binding layer, polyacrylamide (APA) diffusive gel, and HVLP filter were applied for both accumulations as well as the pre-concentration of chemically labile iron compounds in situ in the sediment porewaters of Gradiscica, a river in Slovenia (46°2′38″ N 14°30′20″ E). Two DGT probes were put back-to-back in sediment approximately 6 cm deep for 84 h. The temperature was monitored regularly to apply the appropriate diffusion coefficient.

To find a concentration of iron species in samples collected from the sediment porewater, calibration curves for Fe^2+^ and Fe^3+^ (reduced to Fe^2+^) bounded by Ch-100 and SPR-IDA resin were constructed in a concentration range of 0–1 μM. Calibration gels were prepared by loading the resin with an Fe^2+^ solution, which subsequently reacted with *o*-phenanthroline to form a colored complex [20].

The obtained calibration curves were used to determine the concentrations of total Fe (TFe) and Fe^2+^ in the surrounding medium of the DGT probe as well as their one-dimensional (1D) distributions within the Ch-100 and SPR-IDA resins.

After collecting the samples, the DGT probes were taken out of the sediment, washed out with ddH_2_O, put into a foil bag, and transported to the laboratory. Both the diffusive layer and filter were removed, while the resin layer was taken to a clean vial filled in with ddH_2_O and kept there until analysis was performed. One of the probe gels was further employed to find the dissolved Fe^2+^ concentration, whereas the second probe was used to determine the TFe amount. Then, the gel was dried on a glass support (slide) for further characterization of iron content by the BDS.

The presented method was applied to find the amount of dissolved iron species bounded in Ch-100 or SPR-IDA resin as well as their 1D distribution within these gels after their deployment in sediment porewater. The improvement in sensitivity of the BDS technique, after introduction of the cavity for the PB, was tested on the samples prepared according to the procedure described above.

### 2.5. Method

The Fe^2+^ detection requires the performance of a colorimetric reaction that involves the use of 1.10-phenanthroline as a ligand to provide the formation of a Ferroin complex that is characterized by an intensive red-orange color with an absorption maximum in the visible range of the spectrum (510 nm). The determination of TFe is based on Fe^3+^ conversion to Fe^2+^ by a reducing agent (L-ascorbic acid-AA) before performing the colorimetric reaction.

**Figure 2:**
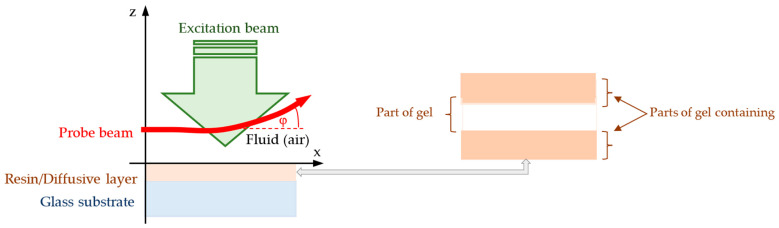
Scheme of the BDS experimental setup.

The scheme of the BDS experiment [67,68] is shown in Figure 2. The photothermal signal carries information about the materials’ thermal parameters (thermal diffusivity and conductivity), which in turn are determined by its chemical composition.

### 2.6. Theory

The temperature oscillations (TOs) *υ_i_* induced in *i*th layer of the analyzed sample (Figure 2 and Appendix A) were found using the Fourier–Kirchhoff equation [67,68]:

(3) $$D_{Ti}^{-1}\frac{\partial \vartheta_{i}}{\partial t}=\nabla^{2}\vartheta_{i}+q_{i}k_{Ti}^{-1}$$

where *q_i_* is the power density of internal heat source; *k_Ti_* and *D_Ti_* are thermal conductivity and diffusivity of the sample’s *i*th layer. The EB incident light is modulated with frequency *f*, which leads to EB intensity variation in a range from 0 to *I_EB_*. The light energy absorbed by the sample induces internal heat sources in each of its layers (Figure 2, Appendix A), given by [67,68]

(4) $$q_{si}\left(x,t\right)=2^{-1}\gamma_{i}I_{EB}exp\left[\sum_{m=1}^{i-1}\gamma_{m}\left(L_{m-1}-L_{m}\right)+\gamma_{i}\left(L_{i-1}-x\right)\right]exp\left(2i\pi ft\right)$$

Here, *γ_i_* and *L_i_* denote the optical absorption coefficient and thickness of the sample’s *i*th layer, respectively. *I_EB_* is the EB intensity lighting the sample’s surface.

After determination of TOs in the layered structure, as presented in Figure 2, a theoretical model of PB intensity change when undergoing the TOs must be found. For that purpose, the theory of complex geometrical optics was used [67,68]. It is based on assumption that the PB can be treated as a bundle of rays that propagates in a complex space. The interaction of the PB with TOs leads to the deviation of rays’ trajectories from their initial direction of propagation, which is called the PB deflection (Appendix A) and is caused by the presence of gradients of refractive index in fluid, where the PB propagates as expressed by

(5) $$∆z\left(\xi ,\tau \right)=n_{0}^{2}s_{T}\int_{0}^{\tau }\left(\tau -\tau \prime \right)\frac{\partial \vartheta_{f}}{\partial z}d\tau \prime$$

where *n*_0_ is the refracting index of undisturbed fluid, *s_T_* = (1/*n*_0_)(*dn*/*dT*) is the temperature coefficient of refractive index (thermal sensitivity), *τ* is the running complex coordinate along the PB trajectory, and *ξ* is the PB’s coordinate in the input plane of the experimental setup (*z* = 0). The PB is assumed to enter the experimental setup in the plane *z* = 0 and propagates in the positive direction of the *OZ* axis.

The PB trajectory change leads to increase in PB beam divergence, which consequently changes PB amplitude (Appendix B) [67]:

(6) $$A\left(z\right)≅A_{0}\left(1+∆a_{1}+∆a_{2}\right)$$

Here, $∆a_{1}$ and $∆a_{2}$ are the corrections to the PB amplitude after its 1st and 2nd pass through TOs, respectively; *A*_0_ = *E*_0_ *z_R_ z_RC_*^−1^ is the amplitude of the undisturbed PB; *E*_0_ is the electric field amplitude of the undisturbed PB in its center; *z_R_* = *ka*^2^*n*_0_ is the distance from the PB center to the point where it radius is doubled (Rayleigh length); *z_RC_* = *z_R_* − *iL*_0_ is the complex Rayleigh length; *L*_0_ is the focal distance; *a* is the radius of the PB in its center; *k* is the PB wave number; and *λ* is its wavelength.

Another effect of PB interaction with TOs is its phase change, expressed as [67]

(7) $$∆\Phi =∆\Phi_{1}+∆\Phi_{2}=kn_{0}^{2}s_{T}\int_{0}^{\tau }\vartheta_{f}\left[z\left(\tau \prime \right)\right]d\tau \prime =∆\Phi_{1d}+∆\Phi_{2d}+∆\Phi_{1f}+∆\Phi_{2f}$$

where $∆\Phi_{1d}$ and $∆\Phi_{2d}$ are changes in the PB phase caused by its deflection on thermal gradients, whereas $∆\Phi_{1f}$ and $∆\Phi_{2f}$ are changes in the PB phase introduced by the change in fluid refractive index in which the PB propagates. Indexes “1” and “2” refer to the 1st and 2nd pass of the PB through TOs (Appendix B).

Finally, the PB intensity variations after passing twice through TOs at the detector $\left(z_{D}\right)$ can be written as

(8) $$I\left(x_{D},y_{D},z_{D}\right)=I_{0g}\left(x_{D},y_{D},z_{D}\right)\left\{1-2kIm\left[∆\Phi_{1}\left(z_{D1}\right)+∆\Phi_{2}\left(z_{D2}\right)\right]\;+2Re\left[∆a_{1}\left(z_{D1}\right)+∆a_{2}\left(z_{D2}\right)\right]\right\}$$

where $∆\Phi_{1}\left(z_{D}\right)$, $∆\Phi_{2}\left(z_{D2}\right)$, and $∆a_{1}\left(z_{D}\right)$, $∆a_{2}\left(z_{D2}\right)$ are described by Equations (6) and (7); $z_{D1}$ is the position of mirror reflecting the PB and directing it the 2nd time through the area of TOs; $z_{D2}$ is the detector position with respect to the position of the reflecting mirror; *I*_0*g*_(*x_D_*, *y_D_*, *z_D_*) is the undisturbed PB intensity, which has the following form:

(9) $$I_{0g}\left(x_{D},y_{D},z_{D}\right)={\left(E_{0}z_{D}{z_{RC}}^{-1}\right)}^{2}{\left|exp\left\{ikn_{0}z_{D}\left[1-\left(x_{D}^{2}+y_{D}^{2}\right){\left(2z_{RC}^{2}\right)}^{-1}{\left(1+iz_{D}{z_{RC}}^{-1}\right)}^{-1}\right]\right\}\right|}^{2}$$

Here, *x_D_*, *y_D_*, and *z_D_* are the coordinates of the ray at the detector plane.

The change in PB intensity introduced by TOs results in a photodeflection (BDS) signal that, for detection performed by quadrant photodiode (QP), can be written as

(10) $$S_{BDS}=2K_{d}\left\{\int_{0}^{+\infty }-\int_{-z_{0}}^{0}\;\right.dx\left.\int_{-\infty }^{+\infty }dy\left[Re\left(∆a\right)-kIm\left(∆\Phi \right)\right]I_{0}\right\}=A_{BDS}cos\left(2\pi ft+atan\left(\Theta_{mI}/\Theta_{mR}\right)+\varphi_{BDS}\right)$$

(11) $$∆a=∆a_{1}\left(z_{D1}\right)+∆a_{2}\left(z_{D2}\right),\;∆\Phi =∆\Phi_{1}\left(z_{D1}\right)+∆\Phi_{2}\left(z_{D2}\right)$$

where *K_d_* is the detector constant, *z*_0_ is the PB height over the sample surface, *A_BDS_* is the change in PB amplitude caused by deflection, and *φ_BDS_* are variations in PB phase resulting from fluid refractive index change because of TOs and deflection of the PB on thermal gradients, respectively.

### 2.7. Experimental Setup

**Figure 3:**
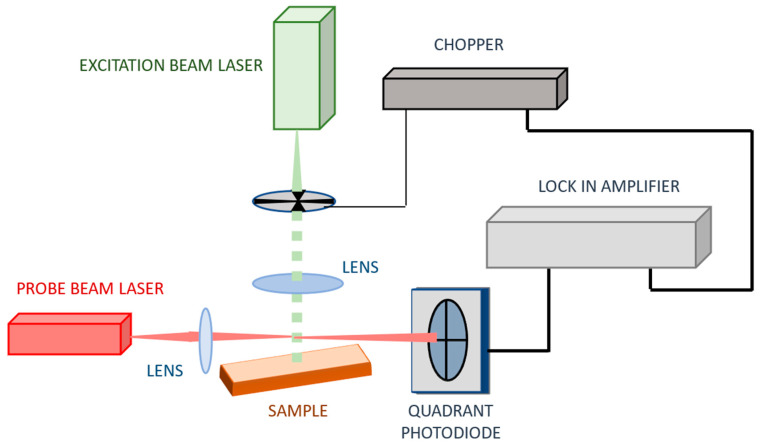
The conventional BDS experimental setup.

**Figure 4:**
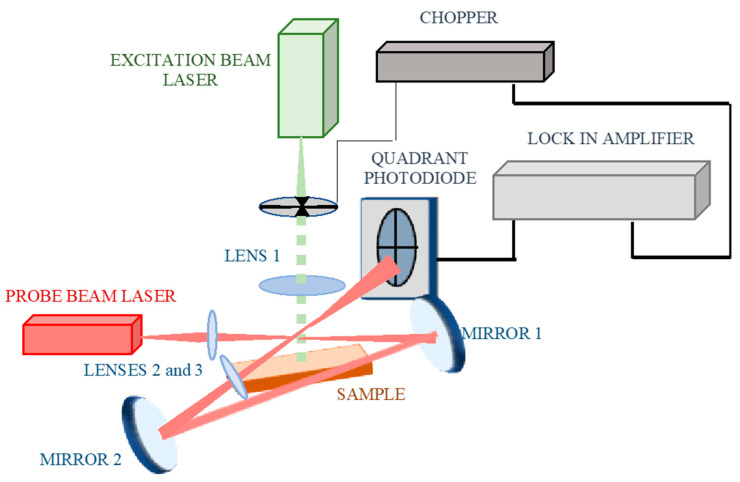
The optimized BDS experimental setup.

The measurements were carried out using both the conventional BDS experimental setup (Figure 3) and the optimized one (Figure 4). As the EB source, a solid-state laser (532 nm, 30 mW) (BWI-532-10-E/66966) was chosen. It was intensity-modulated using a mechanical chopper (SCIENTIC INSTRUMENTS 300CD, 300C, 300H model). The modulation frequency was 3 Hz. The PB was a Helium-Neon laser (633 nm, 3 mW) (Uniphase, Model 1103P, St. Charles, IL, USA). Both laser beams were shaped by bi-convex, AR-coated lenses (350–700 nm, EDMUND OPTICS, Barrington, NJ, USA). The EB was defocused to a spot of 2 mm^2^ in size on the surface of the examined sample by a lens of 100 mm focal length. The size of the EB defines the spatial resolution of the BDS technique. The PB was focused to a size of 40 μm in diameter by a lens of 40 mm focal length. The PB center was settled over the sample. Such arrangements of the BDS setup provide the 1D experimental configuration since the size of PB << EB [69].

The conventional BDS system (Figure 2) was further upgraded by introducing a cavity for the PB (Figure 4). It ensures the direction of the PB twice through area of TOs. Such modification is expected to ensure a higher sensitivity of the BDS setup because the PB intensity change is enhanced by increasing the length of its interaction with TOs.

For this purpose, additional mirrors (broadband, 400–750 nm, Thorlabs, model BB1-E02) were applied. The change in PB intensity was measured by QP (RBM–R. Braumann GmbH, Attenkirchen, Germany, Model C30846E) equipped with interference filter (CWL 633 nm, Edmund Optics, Barrington, NJ, USA). The BDS signal was collected by a lock-in amplifier (Stanford research instruments, Model SR830 DSP, Sunnyvale, CA, USA) connected to a PC. The sample was put onto a translation stage (CVI, Model 2480M/2488) to determined its 3D movement to geometrically optimize the configuration of the BDS system.

### 2.8. Optical Microscope

The images of prepared and dried resins were collected by optical microscope (Olympus IX81 Bruker, Karlsruhe, Germany) using 40× magnitude.

### 2.9. Statistical Analysis

All experiments were performed five times independently. The obtained results from all experiments are expressed as mean ± SD. The analysis of statistical significance was performed using Tukey’s means test (*p* ≤ 0.05).

## 3. Results and Discussion

**Figure 5:**
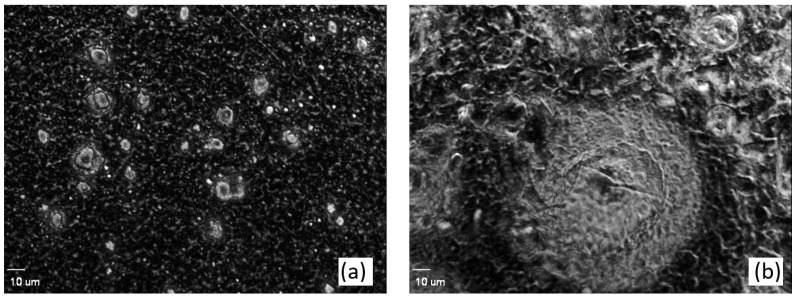
The microscope images of (**a**) SPR-IDA and (**b**) Ch-100 resins.

The images of dried SPR-IDA and Ch-100 resins taken using the optical microscope are presented in Figure 5. It is seen that Ch-100 resin is characterized by a bead size of 100 µm, whereas the SPR-IDA’s bead size is 0.2 µm. The SPR-IDA resin with a smaller bead size is the more homogeneous resin-gel compared to coarser Ch-100 resin.

### 3.1. Optimization of the Sample’s Preparation Protocol

**Figure 6:**
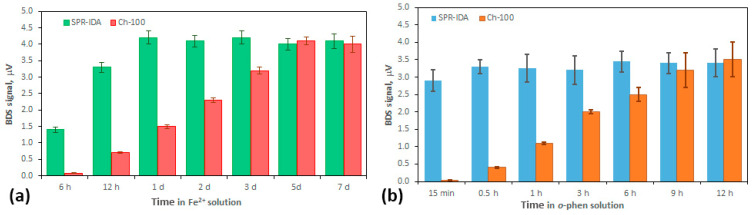
BDS signal measured for SPR-IDA and Ch-100 resins being immersed in (**a**) 1 μM Fe^2+^ solution followed by a reaction with *o-*phen and (**b**) *o-*phen solution having bound 1 μM Fe^2+^. The measurement uncertainty was calculated as the SD of five repetitions of the measurement for different samples containing the same amount of bounded iron ions.

Analysis of the diffusion process of Fe ions into the SPR-IDA and Ch-100 resins used in the DGT sampling technique provides information about the mechanisms of iron transportation within the resin. Such information is essential for optimizing the sampling protocol (defining the proper time for which the sampler should be left in the real water environment) as well as for improving the sensitivity and accuracy of the whole detection technique. Characteristics of the diffusion process depend on many factors, like time of resin immersion in solution, use of a stirring process, and temperature during the experiment. The time of the DGT probes’ immersion refers to the time interval in which the DGT resin layer is in contact with aquatic environment (sample). Thus, in the very first step of the study, it is necessary to determine the complexation rate of the Fe^2+^ reaction with *o-*phen and the formation of a Ferroin complex inside SPR-IDA and Ch-100 resins. For this reason, all gels were put into 25 mL flasks containing Fe^2+^ solutions (C = 1 μM) for an immersion time of 7 days. After that time, the resins were transferred into *o-*phen solution (C = 3 μM) and left for different time periods that varied from 15 min up to 12 h. After these time intervals, the resins were transferred onto a glass slide and left for drying at room temperature. The collected BDS signal as a function of the resin’s immersion time in 1 μM of Fe^2+^ solution is presented in Figure 6a. It is seen that the minimum value of required immersion time is no less than 1 day in the case of SPR-IDA resin and 5 days for Ch-100 to complete the binding process of all Fe^2+^ presented in the solution. It must be mentioned that this time can be significantly decreased (to 1 h (SPR-IDA)/5 h (Ch-100)) when active stirring of the Fe^2+^ solution is provided. The results indicate that the SPR-IDA resin gel exhibits faster saturation than the Ch-100 resin. The reason for this is the different bead sizes of these two types of resins. The Ch-100 bead size is 100 µm, whereas that of SPR-IDA is much smaller and has a value of 0.2 µm. Thus, SPR-IDA resins have a higher active-surface-area-to-volume ratio compared to the Ch-100 resin, which is reflected in the presence of more binding sites for Fe^2+^. Thus, the binding capacity of both resin gels needs to be further tested (Section 3.2).

To optimize the measurements procedure, the time needed for performing the colorimetric reaction of all bounded iron ions with *o*-phen must be found. Thus, the BDS amplitudes as a function of the resin immersion time in *o-*phen solution were collected. The results can be seen in Figure 6b. The *o*-phen has no polar group, which makes it non-hydrophilic; hence, molecules can easily penetrate into the depth of the resin. The exact mechanism of the *o*-phen reaction with the iron ions bounded by the resins has not been found, but it can be attributed to the presence of different functional groups in both resins (paired iminodiacetate ions and iminodiacetate for Ch-100 and SPR-IDA, respectively). This seems to be the reason why various times intervals of resin immersion in *o*-phen are required to complete the formation of Ferroin within both resin gels.

The results indicate that the reaction of complexing Fe^2+^ using *o*-phen inside SPR-IDA and Ch-100 resins is relatively fast (0.5 h (SPR-IDA)/9 h (Ch-100)) and can be still increased by providing an active stirring process (10 min (SPR-IDA)/3 h (Ch-100)).

### 3.2. Cavity for PB

**Figure 7:**
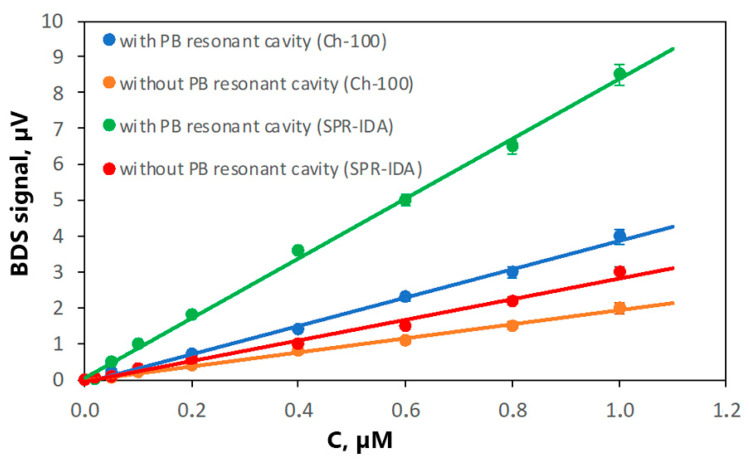
Calibration curves for Fe^2+^ and TFe determination using both SPR-IDA and Ch-100 resins. Points represent the measured data, whereas the solid lines represent the regression lines. The measurement uncertainties are determined as the SD of five repetitions of the measurement for different samples containing the same amount of bounded iron ions.

After optimization of the samples’ preparation procedure, the experimental setup was modified to ensure high sensitivity of the measurements. For that purpose, the cavity for the PB was built to direct the PB twice through the area of induced TOs. The upgraded BDS system was then applied for constructing the calibration curves for Fe^2+^ and TFe determination (Figure 7).

The method’s sensitivity was determined by finding the lowest species concentration in the examined sample that is possible to be measured with the desired accuracy and precision [70]. The slope of the calibration curve can be used to estimate the LOD of the assay and can be found as 3 multiplied by the value of the standard deviation of the blank signal *SD*_0_ calculated for six repetitions of its determinations and further divided by the slope of calibration curve *a*, according to

(12) $$LOD=3\cdot {SD}_{0}a^{-1}$$

LOQ defines a limit of quantification that determines the smallest species concentration in the analyzed sample that can be found with the desired accuracy and repeatability. It is calculated as

(13) $$LOQ=10\cdot {SD}_{0}a^{-1}$$

The obtained characteristics of calibration curves for both types of examined resins are shown in Table 1. It is seen that directing the PB twice through area of TOs results in an increase in the slope of the calibration curve by a factor of two since each pass causes the PB intensity change, which finally leads to doubling the BDS signal without significant change in the measurement noise (the SD of each measured point remains at the same level).

The calculated LODs and LOQs are much smaller compared to the experimental setup without a PB cavity. The increased number of PBs passing through TOs increases the PB intensity change and consequently the BDS signal, which finally leads to improvement in the method’s sensitivity. This is also reflected in the slopes of the calibration curves, whose changes correspond to the variations in LODs and LOQs. For the optimized BDS experimental setup, the obtained LODs were 40 nM and 20 nM for Ch-100 and SPR-IDA resins, respectively. This means that SPR-IDA resin ensures a twice-higher sensitivity of Fe redox species determination compared to Ch-100 resin. This is the consequence of a smaller size of SPR-IDA beads, which in turn makes the resin’s surface smoother and more uniform and limits the TOs dispersion, leading to enhancement in the BDS signal.

The obtained LODs for both cases are much lower compared to those obtained for the spectrophotometric method (1.07 μM; in 10 cm long sample cell) and thermal lens microscopy in combination with microfluidic systems (TLM-μFIA) using 1–5 μL of sample (36 nM) [71], and it was comparable to thermal lens microscopy (TLM) in batch mode (10 nM).

The results indicate that DGT-BDS provides low enough LODs for determining concentrations of iron species in natural aqueous media, where their concentrations often are below 1 μM [18]. Nevertheless, the LOD of the DGT-BDS technique is still much lower than the iron concentrations in ocean waters (0.2 nM) [72,73] and in aqueous media with an oligotrophic environment, where iron is a limiting nutrient for phytoplankton and other primary producers [74,75]. Fortunately, it can be further decreased by increasing the time interval of the sample’s deployment in the water environment, as indicated by Equation (1).

**Figure 8:**
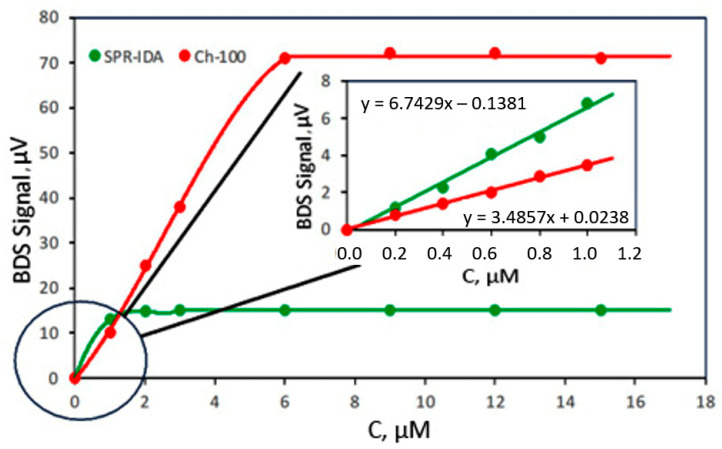
Adsorption capacity of resins within a 1-day immersion time into Fe^2+^ solutions.

It must be also stated that each of the resins has its own saturation limit; thus, both of them are applicable depending on the purpose of their use. In order to determine the binding capacities of Ch-100 and SPR-IDA, both resins were immersed in 25 mL flasks containing Fe^2+^ solutions for 1 day in the concentration range 0–15 μM and then transferred into the solution of *o*-phen. It was found that SPR-IDA gel saturates much faster than Ch-100 (Figure 8). It reached a saturation limit for Fe concentration that was higher than 1 μM. This fact suggests that SPR-IDA resins exhibit much lower binding capacity (1 < μM), which may be further reduced in environments with competing metal ions, such as Cu or Cd. Thus, it cannot be used in the case of highly contaminated sediments but rather in conditions of very low iron concentrations in natural waters, whereas Ch-100 resins are useful in the presence of Fe in a higher concentration range.

### 3.3. Validation of the Method

The validity of the developed method was tested by determining its LOD/LOQ, linearity range, accuracy/precision, stability, and selectivity/specificity [76]. The obtained calibration curves (Figure 8) for Fe^2+^ detection in aquatic media using SPR-IDA and Ch-100 resins indicate that a linear function of the BDS signal’s dependence on Fe^2+^ concentrations was obtained in a concentration range from 0 to 1 μM in the case of SPR-IDA resin and up to 6 μM for Ch-100 resin. The linearity was found by applying the least square regression procedure, and it determines the extent to which the output of the BDS setup reflects the true value of the Fe^2+^/TFe being measured. The value of calculated correlation coefficients (r^2^) was found to be over 0.995. This means that a good linear correlation was obtained for both types of resins.

The relative standard deviation (RSD) of each measured point of calibration lines is ≤10%. This is an indicator of good reproducibility of the developed technique [76]. The calculated LOD/LOQ is 20 ± 1/66 ± 3 nM and 40 ± 2/132 ± 6 for SPR-IDA and Ch-100 resins, respectively. Measurement accuracy is defined as mean recovery (R) and was found for five replicates of six Fe^2+^ standard concentrations. The results can be seen in Table 2. The values of recovery satisfy the guidelines of the EU and US-EPA, which determine values of recovery between 70 and 120% [70]. Measurement precision is defined as the intra-laboratory reproducibility and repeatability of the method. It is expressed as RSD. The obtained values are within the range of 4 to 10%, which satisfies the criteria introduced by EU and US-EPA stating that RSD should be ≤20%. Thus, a good precision of the developed method was achieved.

Comparing the characteristics found for both resins, it can be concluded that the better accuracy was obtained for SPR-IDA resins, especially for a lower range of iron concentrations, whereas precision is at similar level for both cases. The factor influencing such behavior may not only be the more uniform structure of SPR-IDA resin but also its faster saturation.

The sample stability during the measuring procedure is defined as its resistance to any chemical and/or physical changes [70]. Thus, the measurements were repeated 11 days after the sample preparation to determine again the parameters of the previously obtained calibration curve (Table 3).

No significant difference at 0.05 significant level (*p*-value < 0.05) was obtained between slope values and intercepts calculated after 11 days of sample preparation compared to their initial values. Because of this, it is possible to state that good stability of analyzed samples occurred, which also indicates good reliability of the developed method. In the next step, the analytical yield of the method was determined as the ratio of the measured and actually added concentration of the analyte in the examined sample solution (Table 4). The results show that the analytical yield of the method is satisfactory and in agreement with EU directives for acceptable analytical yields [77] within the range of 80–120% for the determination of metals and trace elements in water.

### 3.4. Study of the Diffusion Process of Iron Redox Species into Resins

**Figure 9:**
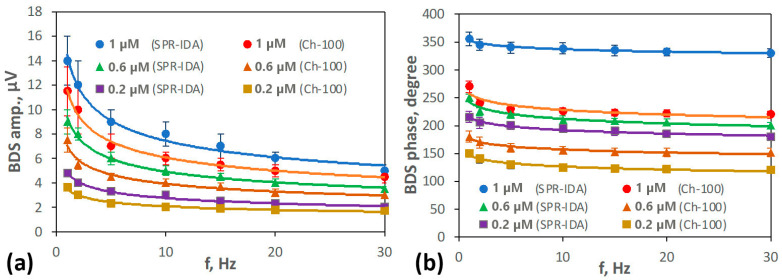
The BDS (**a**) amplitude and (**b**) phase dependence on the modulation frequency of the EB for resins loaded with different concentrations of Fe^2+^ within the 1-day immersion period. Points represent the measured data, whereas the solid lines are the best fit of theoretical dependences. The measurement uncertainties are determined as the SD of five repetitions of the measurement for different samples containing the same amount of bounded iron ions.

After optimizing the sample preparation procedure, the diffusion depth of Fe ions into the resins was found by performing a frequency scan of the SPR-IDA and Ch-100 samples from the bulk to subsurface layer by collecting the BDS signal amplitude and phase with respect to the EB modulation frequency in the range from 1 to 30 Hz (Figure 9). To comply with fitting protocol, both resins were divided into three sub-layers. The thermal properties of each sub-layer were determined to find any changes across the entire resin in its thermal properties that would indicate the values of Fe diffusion depth into it. The criterion for defining sub-layer thickness is a change in the value of thermal diffusivity that equals 3 times the standard deviation in the determination of thermal diffusivity of pure Ch-100/SPR-IDA, which was found to be *SD* = 0.005 mm^2^s^−1^. The analysis consisted of several steps, as described in Appendix C.

The measurements were carried out using Ch-100 and SPR-IDA resins containing different amounts of iron species (in the concentration range of 0–1 μM). The increase in the BDS signal with the increase in Fe concentration can be seen in Figure 9a. The reason for such signal behavior is the enhanced optical absorption by diffusion of Fe species into the resins.

To extract the information about the diffusion depth of different amounts of iron ions into the Ch-100 and SPR-IDA resins, multi-parameter fitting of theoretical BDS signal dependences (Equation (11)) to the experimental data (Figure 9) was performed. The fitted parameters included the thermal diffusivities and conductivities of the Ch-100 and SPR-IDA sub-layers as well as their thicknesses. The thermal properties of the glass support were taken as known parameters (0.34 mm^2^ s^−1^; 0.8 W m^−1^ K^−1^) in the fitting procedure.

**Figure 10:**
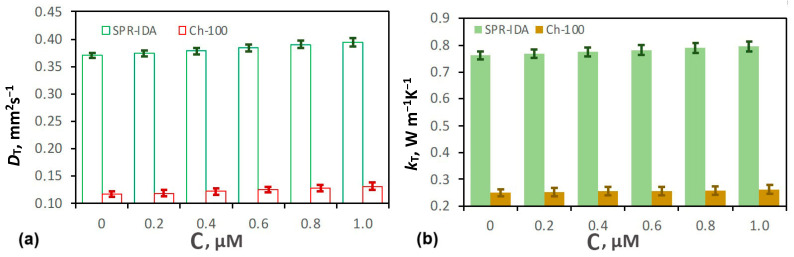
The (**a**) thermal diffusivities *D_T_* and (**b**) conductivities *k_T_* of SPR-IDA/Ch-100 resins containing different concentrations of Fe^2+^. The values were found by performing a fitting procedure of theoretical dependances (Equation (11)) to the experimental data (Figure 9). The uncertainty of parameter determination was calculated as the SD of five repetitions of the fitting procedure for different samples containing the same amount of bounded iron ions.

The thermal diffusivity (*D_T_*) and thermal conductivity (*k_T_*) of pure SPR-IDA/Ch-100 resins was found to be 0.370 ± 0.005/0.117 ± 0.005 mm^2^ s^−1^ and 0.762 ± 0.016/0.250 ± 0.014kW m^−1^ K^−1^, respectively. The thermal properties of both SPR-IDA/Ch-100 resins containing different amounts of iron are shown in Figure 10. It can be seen that both values of *D_T_* and *k_T_* increase with the increase in concentration of iron species. The material’s thermal properties are determined by its molecular structure, which in turn defines how heat is conducted through it and exchanged with the sample’s surroundings. The presence of iron species in the resin enhances its thermal properties. Iron species can introduce additional bonding sites in the resin matrix, which can increase the number of intermolecular interactions and reduce the free volume available for heat transfer. This can increase the thermal diffusivity and conductivity of the whole resin by reducing the time it takes for heat to propagate through the material and exchange with its surroundings, thus reaching the thermal equilibrium. In our case, this simply means that higher thermal properties indicate more iron being bounded by the resin. These results coincide with those presented in Section 3.1 and Section 3.2.

Furthermore, there is a difference in the values of the thermal properties for the two type of analyzed resins. In case of SPR-IDA resin, the values of thermal diffusivities and conductivities are over three times higher compared to those of Ch-100 (Table 5). The reason for this is the smaller bead size of the SPR-IDA resin, which introduces more binding centers for Fe inside the gel and increases the material’s thermal properties. It also masks the influence of internal interfaces and increases the value of thermal resistance.

Thus, by determining the areas of increased/decreased Fe concentrations in SPR-IDA and Ch-100 resins with increased thermal properties resulting from iron bounding, their diffusion depths can be found. This in turn enables the estimation of potential mobility and bioavailability of Fe species in aquatic medium (see Appendix A).

The greater the number of Fe-binding sites available in the resin, the deeper the iron ions can penetrate, increasing the overall amount of bound iron. This makes the SPR-IDA resin more effective and enhances its interaction with iron ions in the aqueous system of interest [61,78]. Consequently, the diffusion depth of Fe into Ch-100 resin is lesser than that of SPR-IDA. This is because of Ch-100’s larger bead size, which reduces the number of Fe binding sites. This makes the Ch-100 resin less reactive and selective for Fe ion determination, as a greater diffusion depth allows for more effective binding and accumulation of iron ions from the surrounding environment rather than just at the outer surface of the resin. The higher selectivity of resins with greater diffusion depths can be attributed to their ability to discriminate between metal ions, as they tend to preferentially bind the target metal, thereby displacing competing ions from the binding sites.

### 3.5. In Situ Application of Ch-100 and SPR-IDA Resins

The applicability of the upgraded BDS-DGT method was tested by deploying the Ch-100 and SPR-IDA resins in the local river sediment (45°56′32.93″ N 13°38′22.92″ E) to determine the vertical concentration of Fe species in the sediment porewater.

After the determination of Fe redox concentrations in the bounding resin, the DGT equation (Equation (1)) was used to calculate the concentration of Fe species in the sediment porewaters. Fe^2+^ and Fe^3+^ concentrations increased sharply across the water–sediment interface (SWI) and remained constant afterwards.

To determine if there was a significant difference between the vertical Fe redox species profiles obtained using probes with SPR-IDA and Ch-100 resins, *p*-values were calculated at the 95% confidence level (95% CI), as described in ref. [79]. If the calculated *p*-value has the value of 0.05 or lower, the difference between compared properties is considered to be statistically significant. In our case, any significant differences in iron vertical distribution could not be confirmed at a 95% CI since *p* > 0.05 for all cases.

**Figure 11:**
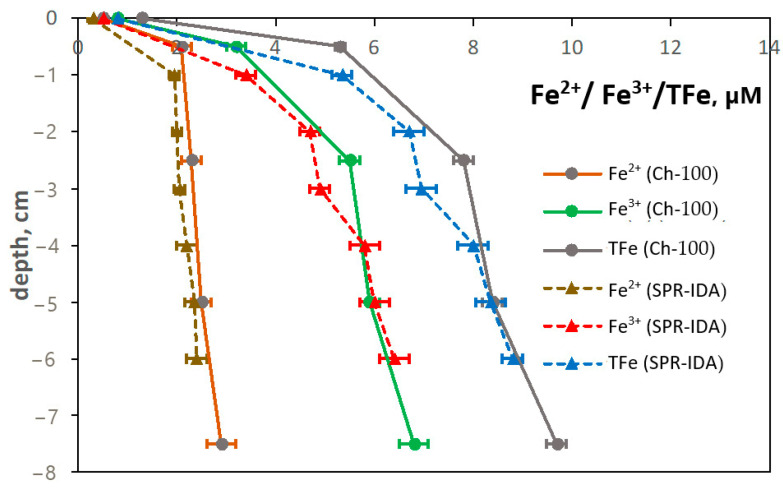
Vertical concentration changes of Fe redox species in sediment porewater in local river (45°56′32.93″ N 13°38′22.92″ E). The measurements uncertainties are determined as the SD of five repetitions of the measurement for different samples containing the same amount of bounded iron ions.

It was found that in three days, averaged TFe, Fe^2+^, and Fe^3+^ concentrations in the sediment porewaters were in the range of 0.3–8.8 μM. These concentrations may represent up to 87 percent of the true sedimentary Fe concentration value, thus excluding the bias of DIFS (DGT-induced fluxes in sediments and soils) through solute resupply [80]. DGT uptake in sediments is generally diffusion-limited, and the observed maxima and minima reflect environmental reality and are not induced by the DGT [21,60,81]. The three-day deployment should be sufficiently long for the sediments to reassemble around the probe and ensure good contact with the probe’s surface and avoid artefacts resulting from a limited contact between the DGT device and the sediment, which causes the intrusion of the overlying water [82]. Furthermore, the size of the individual EB measurement spot takes into account any bias introduced by lateral diffusion of iron species during passage through the diffusive layer. The determined distribution of Fe shown in Figure 11 is therefore considered to be an authentic representation of the natural situation [81,83].

Although the spatial resolution of the DGT-BDS method is less than that of Fe^2+^-specific DET [84], our results show that two back-to-back deployed DGT probes may nonetheless image sedimentary processes on a millimeter scale. The microniche processes are likely to remain undetected by the current setup, which is the limitation of the presented DGT-BDS technique. The sensitivity of the DGT-BDS method is currently limited by the area of measurement, which may theoretically be improved to match the size of SPR-IDA/Ch-100 particles, namely 0.2 μm or 100 μm, respectively.

The concentrations of labile Fe species are within the expected values for porewaters in freshwater sediments [84,85]. Higher Fe^3+^ than Fe^2+^ concentrations indicate generally oxidative conditions within the sediment and imply a deeper oxygen penetration depth [86]. In such a situation, there is a quick oxidation process of Fe^2+^ to Fe^3+^ [87,88]. As a consequence, the microbial activity is limited, which is reflected in the reduction rate of Fe^3+^ to Fe^2+^ [89]. The dominance of Fe^3+^ may also indicate the occurrence of sulfate reduction to sulfide during the anaerobic respiration of microorganisms. This process plays a crucial role in biogeochemical cycles, particularly in environments such as sediments and aquatic ecosystems [86]. Furthermore, comparatively low background values also suggest the absence of a Fe mobilization process, such as sulfate reduction or another microbially mediated reductive metabolism [85].

## 4. Conclusions

In the present study, the BDS technique was optimized by introducing a cavity for PB, coupled to DGT (using two different types of bounding resins) and further applied for the determination of Fe species in an aquatic medium. It was found that the cavity for PB increases the sensitivity of the system, decreasing the LOD of the method by twice compared to the conventional BDS experimental setup.

The obtained LODs were 20 ± 1/40 ± 2 nM for SPR-IDA/Ch-100 resin, which is much lower than values obtained in the case of other spectrometric techniques such as UV–vis spectroscopy (1.07 µM) or thermal lens microscopy (0.01 µM, batch mode) but comparable to LOD values received by thermal lens spectrometry combined with flow injection analysis (36 nM) or thermal lens microscopy coupled to microfluidic systems (36 nM) [90]. SPR-IDA resin was found to have a much lower capacity compared to Ch-100 resin. Thus, in highly contaminated sediments or for long deployment times, the Ch-100 resin should be used as the bounding gel in passive samplers. The application of SPR-IDA resin should then be limited for short deployment times or environments with low iron concentrations. The results also indicate a stable sediment in which processes between iron dissolution, mobilization, and adsorption or precipitation are in balance.

In conclusion, it can be said that Ch-100 resin is characterized by a high capacity for iron binding and thus is useful for determining 1D profiles of vertical ions distribution in highly contaminated aquatic environments where binding competition with other metals ions (e.g., Mn, Cu, and Cd) is present. In such a situation, SPR-IDA resins cannot be applied since these processes further lower the resin capacity. Nevertheless, the smaller bead size of the SPR-IDA resin makes it useful for obtaining high-resolution 2D images of metal distribution in waters and soils as well as studying the finer-scale processes that occur there.

The characteristics of both resins indicate that Ch-100 gels are suitable for studying iron cycling processes and monitoring the accumulation of metal ions in sediments of eutrophic lakes. The SPR-IDA resins can be successfully applied for mapping the distribution of metals in eutrophic environments, which elucidates their interactions with nutrients and other pollutants. Such information is important for understanding iron redox processes and bioavailability [78].

There is not much information available about the direct comparison of both types for resins for their dedicated purpose of environmental sensing. Thus, it is of high importance to understand the resins’ particular strengths and limitations when looking for an appropriate tool for their specific application.

## Figures and Tables

**Table 1:** Comparison of parameters of calibration curves and LODs/LOQs for SPR-IDA/Ch-100 resins after colorimetric reaction with *o*-phen (Ferroin complex) for BDS experimental setup equipped with a cavity for PB and without it.

Type of Gel	Parameters of Calibration Curves		LOD/LOQ, nM	
	With Cavity	Without Cavity	With Cavity	Without Cavity
SPR-IDA	y = 0.008x − 0.049 r^2^ = 0.997	y = 0.003x − 0.050 r^2^ = 0.996	20/66	50/165
Ch-100	y = 0.0038x − 0.005 r^2^ = 0.996	y = 0.002x − 0.029 r^2^ = 0.995	40/132	80/264

**Table 2:** Values of accuracy and precision for Fe^2+^ determination by BDS technique coupled to DGT obtained in the study for different types of binding resins (SPR- IDA and Ch-100) used in the passive sampler.

Type of Gel	_Cnominal_, μM	0.1	0.2	0.4	0.6	0.8	1.0
SPR-IDA/Ch-100	_Ccalculated_, μM	0.118/0.099	0.187/0.196	0.403/0.388	0.594/0.606	0.805/0.773	1.003/0.980
	Accuracy, %	118/99	94/98	101/97	99/100	101/97	100/98
	Precision (RSD), %	10/1	6/3	4/3	5/5	4/4	4/6

**Table 3:** Results of stability examination for Fe^2+^ determination using SPR-IDA and Ch-100 resins containing Fe^2+^ (0–1 mM) in form of Ferroin complex within 11 days of analysis.

Period of Time	1 Day	9 Days	11 Days
	SPR-IDA/Ch-100	SPR-IDA/Ch-100	SPR-IDA/Ch-100
r^2^	0.996/0.994	0.996/0.995	0.996/0.995
Slope, μV nM^−1^	0.0076/0.0027	0.0071/0.0025	0.0076/0.0024
SD, μV L nmol^−1^	0.0003/0.0001	0.0003/0.0003	0.0003/0.0002
Lower limit 95 CI	0.0066/0.0023	0.0062/0.0021	0.0069/0.0021
Upper limit 95 CI	0.0085/0.0031	0.0080/0.0029	0.0083/0.0026
Intercept, μV	0.59/0.0289	0.68/0.0291	0.58/0.0286
SD, μV	0.21/0.0093	0.20/0.0088	0.15/0.0095
Lower limit 95 CI	0.02/0.008	0.12/0.009	0.12/0.007
Upper limit 95 CI	1.17/0.056	1.24/0.057	0.97/0.055

**Table 4:** Comparison of analytical yields of the BDS method coupled to DGT for determination of Fe^2+^ using SPR-IDA and Ch-100 resin.

Added Concentration, μM	Measured Concentration, μM	Yield, %
	SPR-IDA/Ch-100	SPR-IDA/Ch-100
0.1	0.081/0.095	81 ± 8/95 ± 2
0.2	0.176/0.196	88 ± 10/98 ± 3
0.4	0.382/0.384	96 ± 9/ 96 ± 3
0.6	0.577/0.580	96 ± 3/97 ± 4
0.8	0.792/0.784	99 ± 4/98 ± 5
1.0	0.992/0.965	99 ± 3/96 ± 4

**Table 5:** The diffusion depths of SPR-IDA and Ch-100 resins for the different concentration of Fe^2+^.

*C*, μM	SPR-IDA		Ch-100	
	*s*, μm	*D*, mm^2^ s^−1^	*S*, μm	*D*, mm^2^ s^−1^
0	0	0.370 ± 0.005	0	0.117 ± 0.002
0.2	3.5 ± 0.1	0.374 ± 0.006	1.00 ± 0.02	0.119 ± 0.002
0.4	6.3 ± 0.2	0.378 ± 0.006	1.95 ± 0.04	0.122 ± 0.003
0.6	9.6 ± 0.3	0.384 ± 0.006	3.30 ± 0.06	0.125 ± 0.003
0.8	11.8 ± 0.4	0.390 ± 0.007	3.60 ± 0.09	0.126 ± 0.003
1.0	14.0 ± 0.5	0.394 ± 0.008	5.00 ± 0.11	0.131 ± 0.004

**Figure A1:**
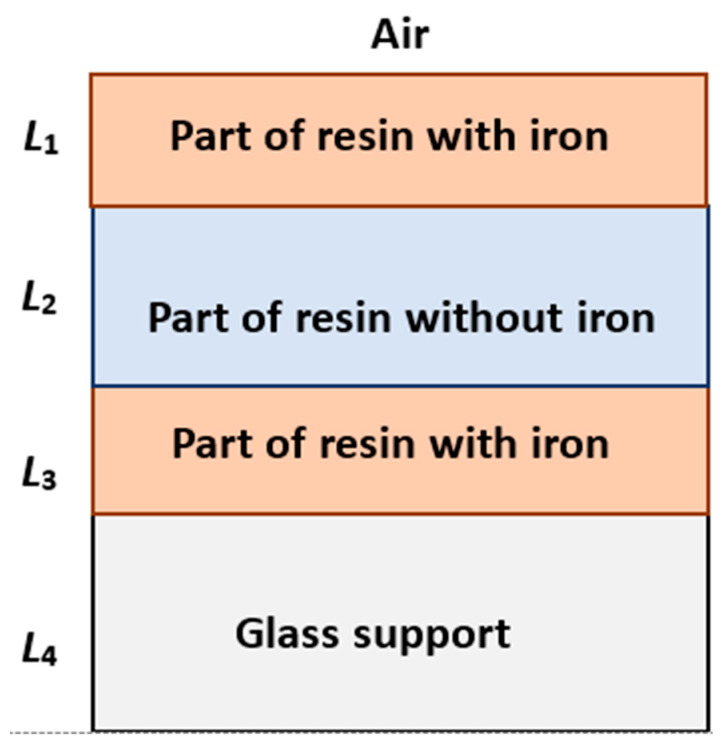
Structure of the sample used in the analysis.

**Figure A2:**
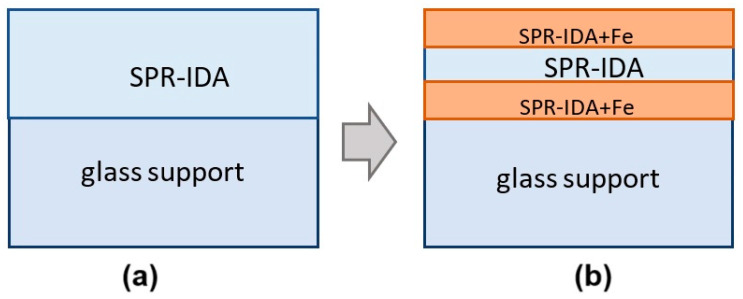
Steps of the fitting procedure: (**a**) collection of the BDS signal from a blank sample (resin without any iron bound in it) deposited on a glass support (two-layered system); (**b**) the BDS analysis performed on four-layered sample consisting of a glass support with bounding resin deposited on it, containing iron layers diffused into it from both its sides (four-layered system).

## Data Availability

Data will be made available on reasonable request from the corresponding author.

## Appendix A

The diffusion of iron ions into the binding resin occurs from both sides of it (Figure A1). Thus, the upper and bottom part of the SPR-IDA resin contain bounded iron ions. The middle part of the resin is its volume that was not reached by the diffusing iron.

The sample of interest is considered to be three-layered (*L*_1_–*L*_3_), with an infinitive high column of air over it, as shown in Figure A1. The absorption of EB radiation occurs only in the layers containing iron ions (*L*_1_, *L*_3_). The absorption coefficients of these layers are *γ*_1_ and *γ*_3_, respectively. The non-radiative de-excitation of the absorbed energy leads to the creation of internal heat sources with a power density of

(A1) $$q_{1}=\frac{I_{0}}{2}\gamma_{1}exp\left[-\gamma_{1}x\right]exp\left[2i\pi ft\right]$$

(A2) $$q_{3}=\frac{I_{0}}{2}\gamma_{3}exp\left[\sum_{m=n}^{2}\gamma_{m}\left(L_{m-1}-L_{m}\right)+\gamma_{3}\left(L_{2}-x\right)\right]exp\left[2i\pi ft\right]$$

where *f* is the modulation frequency of EB.

The solution of Equation (2) with the boundary condition of temperature and heat flux continuity at each layer interface yields the TOs in the air over the sample in the following form:

(A3) $$\vartheta_{g}\left(z,t\right)=\sqrt{\Theta_{mR}^{2}+\Theta_{mI}^{2}}cos\left[atan\left(\Theta_{mI}/\Theta_{mR}\right)+2\pi ft\right]$$

where *D_TG_* is the thermal diffusivity of air, and

(A4) $$\Theta_{mI}=IM\left(\Theta_{m}\right),\;\Theta_{mR}=RE\left(\Theta_{m}\right),\;\Theta_{m}=U_{1}+V_{1}-E_{1}$$

(A5) $$k_{g}=\sqrt{\frac{i\omega }{D_{Tg}}},\;k_{1}=\sqrt{\frac{i\omega }{D_{T1}}},\;k_{2}=\sqrt{\frac{i\omega }{D_{T2}}},\;k_{3}=\sqrt{\frac{i\omega }{D_{T3}}},\;k_{4}=\sqrt{\frac{i\omega }{D_{T4}}}$$

$$V_{1}=D_{1}-D_{3}-\frac{U_{2}+V_{2}}{D_{2}},\;V_{2}=\frac{U_{3}+V_{3}+F_{2}}{F_{1}},\;V_{3}=\frac{U_{4}+V_{4}+G_{5}}{G_{4}},$$

$$V_{4}=\frac{P_{3\;}}{{H_{5}L}_{1\;}}-\frac{P_{2\;}}{{H_{5}L}_{1\;}}\left[\frac{M_{4}}{\left(1-M_{3\;}\right)M_{1\;}}+\frac{M_{1\;}}{M_{2\;}}\right]+\frac{M_{4}}{\left(1-M_{3\;}\right){H_{5}M}_{1\;}}+\frac{M_{1\;}}{H_{5}M_{2\;}}+\frac{H_{4\;}}{H_{5}}$$

(A6) $$V_{5}=\frac{M_{4}}{(1-M_{3\;})M_{1\;}}+\frac{M_{1\;}}{M_{2\;}}$$

$$U_{1}=D_{3}+\frac{U_{2}+V_{2}}{D_{2}},\;U_{2}=\frac{D_{5}+D_{6}}{D_{4}}V_{2},U_{3}=\frac{G_{3}-G_{2}}{G_{1}}V_{3},$$

$$U_{4}=K_{1}+\frac{{K_{2}P}_{3\;}}{P_{1\;}}-\left[\frac{M_{4}}{\left(1-M_{3\;}\right)M_{1\;}}+\frac{M_{1\;}}{M_{2\;}}\right]\frac{{K_{2}P}_{2\;}}{P_{1\;}}+\frac{K_{3}M_{4}}{\left(1-M_{3\;}\right)M_{1\;}}+\frac{{K_{3}M}_{1\;}}{M_{2\;}}$$

(A7) $$U_{5}=\frac{P_{3\;}}{P_{1\;}}+\left[\frac{M_{4}}{(1-M_{3\;})M_{1\;}}+\frac{M_{1\;}}{M_{2\;}}\right]\frac{P_{2\;}}{P_{1\;}},\;U_{6}=\frac{M_{4}}{1-M_{3}}$$

$$M_{1\;}=exp\left[-k_{2}\left(L_{2\;}-L_{3}\right)\right]-\frac{P_{2\;}}{P_{1\;}}exp\left[k_{3}\left(L_{2\;}-L_{3\;}\right)\right],$$

$$M_{2\;}=E_{3}exp\left(C_{2\;}-{\gamma_{3\;}L}_{3\;}\right)-\frac{P_{3\;}}{P_{1\;}}exp\left[k_{5}\left(L_{2\;}-L_{3\;}\right)\right]$$

(A8) $$M_{3\;}=-\frac{P_{2\;}}{P_{1\;}m_{1\;}},\;M_{4\;}=\frac{P_{3\;}}{P_{1\;}}-\frac{P_{2\;}}{P_{1\;}}\frac{m_{1\;}}{m_{2\;}}$$

$$P_{1\;}=\frac{K_{4\;}}{H_{5\;}}exp\left[{-k}_{2}\left(L_{1}-L_{2\;}\right)\right]-k_{1}k_{2}exp\left[k_{2}\left(L_{1}-L_{2\;}\right)\right]+\frac{k_{T3\;}}{k_{T2\;}}\;k_{2},$$

$$P_{2\;}=\frac{k_{2\;}}{H_{5\;}}exp\left[{-k}_{2}\left(L_{1}-L_{2\;}\right)\right]-k_{2}k_{1}exp\left[k_{2}\left(L_{1}-L_{2\;}\right)\right]-\frac{k_{T3\;}}{k_{T2\;}}k_{3},$$

(A9) $$P_{3\;}=k_{2}k_{1}exp\left[k_{2}\left(L_{1}-L_{2\;}\right)\right]-\frac{H_{4\;}}{H_{5\;}}k_{2}exp\left[{-k}_{2}\left(L_{1}-L_{2\;}\right)\right]-\frac{k_{T3\;}}{k_{T2\;}}E_{3}exp\left(C_{2\;}-{\gamma_{1\;}L}_{2\;}\right)$$

(A10) $$K_{1}=\frac{H_{3}}{H_{1}}-\frac{H_{2}H_{4}}{H_{1}H_{5}},\;K_{2}=-\frac{H_{2}}{H_{1}H_{5}},K_{3}=-\frac{H_{2}}{H_{1}H_{5}}$$

$$H_{1}=k_{1}\left(\frac{G_{2}}{G_{1}G_{3}}+\frac{1}{G_{4}}\right)+k_{2}\frac{k_{T2}}{k_{T1\;}},\;H_{2}=k_{1}\left(\frac{G_{2}}{G_{1}G_{3}}+\frac{1}{G_{4}}\right)-k_{2}\frac{k_{T2}}{k_{T1\;}},\;H_{3}=k_{1}\left(G_{6}-1\right)$$

$$H_{4}=-E_{3}exp\left({C_{2}-\gamma }_{3}L_{2}\right)-\frac{H_{3}}{H_{1}}exp\left[k_{2}\left(L_{1}-L_{2\;}\right)\right],$$

(A11) $$H_{5}=exp\left[{-k}_{2}\left(L_{1}-L_{2\;}\right)\right]-\frac{H_{2}}{H_{1}}exp\left[k_{2}\left(L_{1}-L_{2\;}\right)\right]$$

$$G_{1}=k_{1}\left(1+\frac{1}{F_{1\;}}-\frac{D_{5}}{{D_{4}F}_{1\;}}\right).\;G_{2}=\frac{k_{1}}{F_{1\;}}\left\{1-\frac{D_{5}}{D_{4}}\right\}$$

$$G_{3}=\left\{{\gamma_{1}E}_{1\;}exp\left({C_{2}-\gamma }_{1}L_{1}\right)+k_{1}F_{2}-k_{1}\frac{F_{2}}{F_{1\;}}-{\gamma_{1}E}_{2}exp\left({C_{1}-\gamma }_{1}L_{1}\right)\right\}$$

(A12) $$G_{4}=1-\frac{G_{2}}{G_{1}},\;G_{5}=E_{1}exp\left({C_{2}-\gamma }_{1}L_{1}\right)-\frac{G_{3}}{G_{1}},\;G_{6}=\frac{G_{1}}{G_{3}}-\frac{G_{2}G_{5}}{G_{1}G_{4}}$$

$$F_{1}=\frac{D_{5}}{D_{4}}+1,\;F_{2}=E_{2}exp\left({C_{1}-\gamma }_{1}L_{1}\right)-E_{3}exp\left({{2C}_{1}-\gamma }_{1}L_{1}\right)-\frac{D_{6}}{D_{4}},$$

(A13) $$F_{3}=\frac{D_{6}}{D_{4}}+\frac{D_{5}F_{2}}{D_{4}F_{1}}$$

(A14) $$E_{1}=\frac{\gamma_{1}I_{0}}{{2k}_{T1}\left(\gamma_{1}^{2}-\frac{i\omega }{D_{T1}}\right)},\;E_{3}=\frac{\gamma_{3}I_{0}}{{2k}_{T3}\left(\gamma_{3}^{2}-\frac{i\omega }{D_{T3}}\right)}$$

$$D_{1}=E_{1}\left(k_{g}\frac{k_{Tg}}{k_{T1}}-\gamma_{1}\right){\left(k_{g}\frac{k_{Tg}}{k_{T1}}-k_{1}\right)}^{-1},$$

$$D_{2}=exp\left(-k_{1}L_{1}\right)-exp\left(k_{1}L_{1}\right),$$

$$D_{3}=E_{1}exp\left(-k_{1}L_{1}\right)-E_{2}exp\left({C_{1}-\gamma }_{1}L_{1}\right)-D_{1}exp\left(k_{1}L_{1}\right),$$

$$D_{4}=k_{1}-\frac{k_{1}}{D_{2}}\left[exp\left(-k_{1}d_{1}\right)-exp\left(k_{1}d_{1}\right)\right],$$

$$D_{5}=\frac{k_{1}}{D_{2}}\left[exp\left(-k_{1}d_{1}\right)-exp\left(k_{1}d_{1}\right)\right]{\;+\;k}_{2}$$

(A15) $$D_{6}=\gamma_{1}E_{1}exp\left({C_{1}-\gamma }_{1}L_{1}\right)+k_{1}D_{3}exp\left(-k_{1}L_{1}\right)-k_{1}D_{1}exp\left(k_{1}L_{1}\right)+k_{1}D_{3}exp\left(k_{1}L_{1}\right)$$

(A16) $$C_{1}=2\gamma_{1}L_{1},\;C_{2}=\gamma_{1}L_{1}+\gamma_{3}L_{2}$$

(A17) $$B_{0}=\frac{I_{0}}{2}\gamma_{1},\;B_{1}=\frac{I_{0}}{2}\gamma_{5}exp\left({{-\gamma_{1}L}_{1}+\gamma }_{3}L_{2}\right)$$

## Appendix B

Δ*a*_1_ is a correction resulting from PB deflection on refractive index gradients after its first pass through TOs, described as [67].

(A18) $$∆a_{1}=-{\left(2n_{0}^{2}\right)}^{-1}\left(z_{D1}-z_{s1}\right)\left(z_{p1}-z_{l1}\right){\left(1+iz_{D}z_{RC}^{-1}\right)}^{-1}\frac{\partial P\left(\xi \right)}{\partial \xi }$$

(A19) $$P\left(\xi \right)=\sqrt{2}n_{0}^{2}s_{T}k_{TO}\sqrt{\Theta_{mR}^{2}+\Theta_{mI}^{2}}exp\left[-\sqrt{\pi f/D_{Tg}}z_{0}\left(\tau_{s0}\right)\right]sin\left[2\pi ft-\sqrt{\pi f/D_{Tg}}z_{0}\left(\tau_{s0}\right)+atan\left(\Theta_{mI}/\Theta_{mR}\right)-\pi /4\right]$$

(A20) $$z_{0}\left(\tau_{s0}\right)=\xi \left(1+z_{RC}^{-1}\tau_{s0}n_{0}\right),\;\tau_{s0}=\left(z_{p1}+z_{l1}\right){\left(2n_{0}\right)}^{-1}=z_{s1}/2$$

where *D_Tg_* is the thermal diffusivity of air above the sample surface; *z_l_*_1_ and *z_p_*_1_ are positions of left- and right-illuminated sample’s edges with respect to the input of the experimental system; *k_TO_* is the TOs wavenumber, *ξ* is the PB ray coordinate in the input plane of the experimental system (*z* = 0); *z_RC_* = *z_R_ − iL*_0_ is the complex Rayleigh’s length; *z_R_* = *ka*^2^*n*_0_ is Rayleigh’s length; *a* is PB radius in its center and *L*_0_ its center position; *k* is the PB wave number; *z_D_*_1_ is the detector position; *Θ_mR_* and *Θ_mI_* are described by Equations (A3) and (A4) (see Appendix A) [67].

The consequence of the change in the PB optical path (caused by PB deflection on thermal gradients and by a change in the fluid refractive index in which PB propagates) is the change in PB phase that, after the first pass through TOs, is expressed as [67]

(A21) $$∆\Phi_{1}=kn_{0}^{2}s_{T}\int_{0}^{\tau }\vartheta_{f}\left[z\left(\tau \prime \right)\right]d\tau \prime =∆\Phi_{1d}+∆\Phi_{1f}$$

where $∆\Phi_{1d}$ is the change in the PB phase caused by PB deflection on thermal gradients, whereas $∆\Phi_{1f}$ is the change in the PB phase introduced by the change in the air refractive index in which PB travels.

At the position of the reflecting mirror (*z_D_*_1_), $∆\Phi_{d1}$ and $∆\Phi_{f1}$ are written as

(A22) $$∆\Phi_{1d}=z_{D1}{\left(n_{0}z_{RC}^{2}\right)}^{-1}\left(z_{D1}-z_{s}\right)\left(z_{p}-z_{l}\right)P\left(\xi_{D0},\tau_{s0}\right){\left(1-iz_{D}z_{RC}^{-1}\right)}^{-2}$$

(A23) $$∆\Phi_{1f}=n_{0}s_{T}\sqrt{\Theta_{mR}^{2}+\Theta_{mI}^{2}}\left(z_{p}-z_{l}\right)exp\left[-k_{TO}z\left(\xi_{D0},\tau_{s0}\right)\right]cos\left[2\pi ft-k_{TO}z\left(\xi_{D0},\tau_{s0}\right)+atan\left(\Theta_{mI}/\Theta_{mR}\right)\right]$$

where *ξ_D_*_0_ = *x_D1_*(1 + *iz_D1_z_RC_*)^−1^ is the ray’s coordinate in the input plane of an experimental system for a given observation point, whereas $\tau_{s0}$ is the running coordinate of undisturbed PB. *z_p_* and *z_l_* determine the position of right and left sample’s edge, respectively; *z_s_* = (*z_p_* − *z_l_*)/2; $k_{TO}$ is the TOs wave number [67].

Finally, the PB intensity variations after one pass through TOs at the reflecting mirror can be written as

(A24) $$I_{1}\left(x_{D1},y_{D1},z_{D1}\right)\sim {\left|U\left(x_{D1},y_{D1},z_{D1}\right)\right|}^{2}=I_{0g}\left(x_{D1},y_{D1},z_{D1}\right)\left[1-2kIm∆\Phi_{1}\left(z_{D1}\right)+2Re∆a_{1}\left(z_{D1}\right)\right]$$

where *U*(*x_D_*_1_, *y_D_*_1_, *z_D_*_1_) is the, PB electric field distribution and *I*_0*g*_(*x_D1_*, *y_D1_*, *z_D1_*) is the undisturbed PB intensity, which has the following form:

(A25) $$I_{0g}\left(x_{D1},y_{D1},z_{D1}\right)={\left(E_{0}z_{D1}{z_{RC}}^{-1}\right)}^{2}{\left|exp\left\{ikn_{0}z_{D1}\left[1-\left(x_{D1}^{2}+y_{D1}^{2}\right){\left(2z_{RC}^{2}\right)}^{-1}{\left(1+iz_{D1}{z_{RC}}^{-1}\right)}^{-1}\right]\right\}\right|}^{2}$$

Here, *x_D_*_1_, *y_D_*_1_, and *z_D_*_1_ are the coordinates of the ray at the reflecting mirror plane.

Opposite to the conventional BDS setup (Figure 3), the optimized BDS experimental setup contains a cavity for PB, making it under-pass the TOs twice (Figure 4). In such a situation, the total change in PB intensity introduced by interaction with TOs is

(A26) $$I_{2}\left(x_{D},y_{D},z_{D}\right)=I_{0g}\left(x_{D2},y_{D2},z_{D2}\right)\left\{1-2kIm\left[∆\Phi_{1}\left(z_{D1}\right)+∆\Phi_{2}\left(z_{D2}\right)\right]+2Re\left[∆a_{1}\left(z_{D1}\right)+∆a_{2}\left(z_{D2}\right)\right]\right\}$$

where $∆\Phi_{1}\left(z_{D}\right)$ and $∆a_{1}\left(z_{D}\right)$ are described by Equations (6) and (9), while $∆a_{2}\left(z_{D2}\right)$ and $∆\Phi_{2}\left(z_{D2}\right)$ are found by Equations (6) and (7) for the input plane of the experimental setup defined by the second reflecting mirror (mirror 2 in Figure 4).

## Appendix C

The BDS analysis, used to determine the distribution of iron in SPR-IDA resins, was first performed on a blank sample (SPR-IDA resin without any iron bound in it) deposited on a glass support (two-layered system) (Figure A2a) by the use of frequency scanning method, as described in experimental section. In the second step, the BDS analysis was performed on four-layered sample consisting of a glass support with SPR-IDA resin deposited on it, containing iron layers diffused into it from both its sides (four-layered system) (Figure A2b). In such a way, the thermal properties and thicknesses of both SPR-IDA layers containing different amounts of iron were found (Table 4).

## References

[1] Yu J., Jiao R., Sun H., Xu H., He Y., Wang D. (2022). Removal of Microorganic Pollutants in Aquatic Environment: The Utilization of Fe(VI). J. Environ. Manag..

[2] Measures C.I., Vink S. (1999). Seasonal Variations in the Distribution of Fe and Al in the Surface Waters of the Arabian Sea. Deep Sea Res. Part II Top. Stud. Oceanogr..

[3] Tessier A., Couillard Y., Campbell P.G.C., Auclair J.C. (1993). Modeling Cd Partitioning in Oxic Lake Sediments and Cd Concentrations in the Freshwater Bivalve Anodonta Grandis. Limnol. Oceanogr..

[4] Morel F.M.M., Hudson R.J.M. (1985). The Geobiological Cycle of Trace Elements in Aquatic Systems: Redfield Revisited. Chemical Processes in Lakes.

[5] Gregg W.W., Ginoux P., Schopf P.S., Casey N.W. (2003). Phytoplankton and Iron: Validation of a Global Three-Dimensional Ocean Biogeochemical Model. Deep Sea Res. Part II Top. Stud. Oceanogr..

[6] Mejia J., Roden E.E., Ginder-Vogel M. (2016). Influence of Oxygen and Nitrate on Fe (Hydr)Oxide Mineral Transformation and Soil Microbial Communities during Redox Cycling. Environ. Sci. Technol..

[7] Laufer-Meiser K., Michaud A.B., Maisch M., Byrne J.M., Kappler A., Patterson M.O., Røy H., Jørgensen B.B. (2021). Potentially Bioavailable Iron Produced through Benthic Cycling in Glaciated Arctic Fjords of Svalbard. Nat. Commun..

[8] Wang D.H., Zhang H.H., Lin J.W., Zhan Y.H., He S.Q., Liang S.J., Ji Y., Xi X.Q. (2018). Adsorption of Phosphate from Aqueous Solutions on Sediments Amended with Magnetite-Modified Zeolite. Huanjing Kexue/Environ. Sci..

[9] Li X., Di W., Weiwei M., Wenjun L., Tie L., Maoxu Z. (2019). Kinetic Characterization of Reactivity of Iron Dissolution and Phosphorus Release in Surface Sediments of the Changjiang (Yangtze) River Estuary and the Adjacent East China Sea. Haiyang Xuebao.

[10] Yang W.-B., Tang H., Han C., Ding S.M. (2016). Distribution of Iron Forms and Their Correlations Analysis with Phosphorus Forms in the Sedimentary Profiles of Taihu Lake. Zhongguo Huanjing Kexue/China Environ. Sci..

[11] Xing W., Liu G. (2011). Iron Biogeochemistry and Its Environmental Impacts in Freshwater Lakes. Fresenius Environ. Bull..

[12] Viana L.F., Crispim B.d.A., Sposito J.C.V., Melo M.P.d., Francisco L.F.V., Nascimento V.A.d., Barufatti A. (2021). High Iron Content in River Waters: Environmental Risks for Aquatic Biota and Human Health. Ambient. Agua Interdiscip. J. Appl. Sci..

[13] Laufer K., Nordhoff M., Røy H., Schmidt C., Behrens S., Jørgensen B.B., Kappler A. (2016). Coexistence of Microaerophilic, Nitrate-Reducing, and Phototrophic Fe(II) Oxidizers and Fe(III) Reducers in Coastal Marine Sediment. Appl. Environ. Microbiol..

[14] Scholtysik G., Goldhammer T., Arz H.W., Moros M., Littke R., Hupfer M. (2022). Geochemical Focusing and Burial of Sedimentary Iron, Manganese, and Phosphorus during Lake Eutrophication. Limnol. Oceanogr..

[15] Dittrich M., Wehrli B., Reichert P. (2009). Lake Sediments during the Transient Eutrophication Period: Reactive-Transport Model and Identifiability Study. Ecol. Modell..

[16] Khaled A., Zhang M., Ervens B. (2022). The Number Fraction of Iron-Containing Particles Affects OH, HO 2 and H 2 O 2 Budgets in the Atmospheric Aqueous Phase. Atmos. Chem. Phys..

[17] Dong Y., Xu J., Yue G. (2022). Speciation Study of the Aqueous Fe-Cu-As-Sb-Bi-H2SO4 System and Prediction of Redox Potential in Copper Electrorefining from 25 °C to 70 °C. Chem. Eng. Sci..

[18] Moore J.K., Braucher O. (2007). Observations of Dissolved Iron Concentrations in the World Ocean: Implications and Constraints for Ocean Biogeochemical Models. Biogeosci. Discuss..

[19] Guan D.-X., He S.-X., Li G., Teng H.H., Ma L.Q. (2022). Application of Diffusive Gradients in Thin-Films Technique for Speciation, Bioavailability, Modeling and Mapping of Nutrients and Contaminants in Soils. Crit. Rev. Environ. Sci. Technol..

[20] Zhou C., Gao Y., Gaulier C., Luo M., Zhang X., Bratkic A., Davison W., Baeyens W. (2020). Advances in Understanding Mobilization Processes of Trace Metals in Marine Sediments. Environ. Sci. Technol..

[21] Gao Y., van de Velde S., Williams P.N., Baeyens W., Zhang H. (2015). Two-Dimensional Images of Dissolved Sulfide and Metals in Anoxic Sediments by a Novel Diffusive Gradients in Thin Film Probe and Optical Scanning Techniques. TrAC Trends Anal. Chem..

[22] Canfranc E., Abarca A., Sierra I., Marina M. (2001). Determination of Iron and Molybdenum in a Dietetic Preparation by Flame AAS after Dry Ashing. J. Pharm. Biomed. Anal..

[23] Mohamed R.A., Abdel-Lateef A.M., Mahmoud H.H., Helal A.I. (2012). Determination of Trace Elements in Water and Sediment Samples from Ismaelia Canal Using Ion Chromatography and Atomic Absorption Spectroscopy. Chem. Speciat. Bioavailab..

[24] Ramezanpour M., Raeisi S.N., Shahidi S.A., Ramezanpour S. (2020). Trace Analysis of Pb(II) in Milk Samples by Fe_3_O_4_@SiO_2_@3-chloropropyltriethoxysilane@o-phenylendiamine Nanoparticles as an Unprecedented Adsorbent for Magnetic Dispersive Solid Phase Extraction. Micro Nano Lett..

[25] Francisco B.B.A., Brum D.M., Cassella R.J. (2015). Determination of Metals in Soft Drinks Packed in Different Materials by ETAAS. Food Chem..

[26] Bianchi F., Careri M., Maffini M., Mangia A., Mucchino C. (2003). Use of Experimental Design for Optimisation of the Cold Plasma ICP-MS Determination of Lithium, Aluminum and Iron in Soft Drinks and Alcoholic Beverages. Rapid Commun. Mass Spectrom..

[27] Raju O.V., Prasad P., Varalakshmi V., Reddy Y. (2014). Determination of Heavy Metals in Ground Water By Icp-Oes in Selected Coastal Area of Spsr Nellore District, Andhra Pradesh, India. Int. J. Innov. Res. Sci. Eng. Technol..

[28] Sarzanini C. (1999). Liquid Chromatography: A Tool for the Analysis of Metal Species. J. Chromatogr. A.

[29] Rivera S.M., Canela-Garayoa R. (2012). Analytical Tools for the Analysis of Carotenoids in Diverse Materials. J. Chromatogr. A.

[30] Proch J., Niedzielski P. (2021). Iron Species Determination by High Performance Liquid Chromatography with Plasma Based Optical Emission Detectors: HPLC–MIP OES and HPLC–ICP OES. Talanta.

[31] Tangen G., Wickstrøm T., Lierhagen S., Vogt R., Lund W. (2002). Fractionation and Determination of Aluminum and Iron in Soil Water Samples Using SPE Cartridges and ICP-AES. Environ. Sci. Technol..

[32] Beltrán B.G., Ramos-Sanchez V., Chávez-Flores D., Rodríguez-Maese R., Palacio E. (2020). Total Reflection X-Ray Fluorescence Spectroscopy (TXRF) Method Validation: Determination of Heavy Metals in Dietary Supplements. J. Chem..

[33] Borgese L., Zacco A., Bontempi E., Pellegatta M., Vigna L., Patrini L., Riboldi L., Rubino F.M., Depero L.E. (2010). Use of Total Reflection X-Ray Fluorescence (TXRF) for the Evaluation of Heavy Metal Poisoning Due to the Improper Use of a Traditional Ayurvedic Drug. J. Pharm. Biomed. Anal..

[34] Fagbenro A.A., Yinusa T.S., Ajekiigbe K.M., Oke A.O., Obiajunwa E.I. (2021). Assessment of Heavy Metal Pollution in Soil Samples from a Gold Mining Area in Osun State, Nigeria Using Proton-Induced X-Ray Emission. Sci. Afr..

[35] Das S., Ram S.S., Sudarshan M., Chakraborty A., Thatoi H.N. (2019). Proton-Induced X-Ray Analysis of Soil and Water Samples From Chromite Mine Environment for Determination of Toxic Metal Ion Contamination. J. Appl. Spectrosc..

[36] Wygant B.R., Lambert T.N. (2022). Thin Film Electrodes for Anodic Stripping Voltammetry: A Mini-Review. Front. Chem..

[37] Segura R., Toral M.I., Arancibia V. (2008). Determination of Iron in Water Samples by Adsorptive Stripping Voltammetry with a Bismuth Film Electrode in the Presence of 1-(2-Piridylazo)-2-Naphthol. Talanta.

[38] Yang M., Huang J., Fan J., Du J., Pu K., Peng X. (2020). Chemiluminescence for Bioimaging and Therapeutics: Recent Advances and Challenges. Chem. Soc. Rev..

[39] Seitz W.R., Hercules D.M. (1972). Determination of Trace Amounts of Iron(II) Using Chemiluminescence Analysis. Anal. Chem..

[40] Bernardo-Bermejo S., Sánchez-López E., Castro-Puyana M., Marina M.L. (2020). Chiral Capillary Electrophoresis. TrAC Trends Anal. Chem..

[41] Wilson J.M., Carbonaro R.F. (2011). Capillary Electrophoresis Study of Iron(II) and Iron(III) Polyaminocarboxylate Complex Speciation. Environ. Chem..

[42] Karami C., Alizadeh A., Taher M.A., Hamidi Z., Bahrami B. (2016). UV-Visible Spectroscopy Detection of Iron(III) Ion on Modified Gold Nanoparticles With a Hydroxamic Acid. J. Appl. Spectrosc..

[43] Chen W.-H., Maheshwaran S., Park Y.-K., Ong H.C. (2024). Iron-Based Electrode Material Composites for Electrochemical Sensor Application in the Environment: A Review. Sci. Total Environ..

[44] Souza A., Brandao G., Dossantos W., Lemos V., Ganzarolli E., Bruns R., Ferreira S. (2007). Automatic On-Line Pre-Concentration System Using a Knotted Reactor for the FAAS Determination of Lead in Drinking Water. J. Hazard. Mater..

[45] Tautkus S., Steponeniene L., Kazlauskas R. (2004). Determination of Iron in Natural and Mineral Waters by Flame Atomic Absorption Spectrometry. J. Serbian Chem. Soc..

[46] Kell P., Sidhu R., Qian M., Mishra S., Nicoli E.-R., D’Souza P., Tifft C.J., Gross A.L., Gray-Edwards H.L., Martin D.R. (2023). A Pentasaccharide for Monitoring Pharmacodynamic Response to Gene Therapy in GM1 Gangliosidosis. eBioMedicine.

[47] Pujol L., Evrard D., Groenen-Serrano K., Freyssinier M., Ruffien-Cizsak A., Gros P. (2014). Electrochemical Sensors and Devices for Heavy Metals Assay in Water: The French Groups’ Contribution. Front. Chem..

[48] Bard A.J., Faulkner L.R., White H.S. (2022). Electrochemical Methods: Fundamentals and Applications.

[49] Kudr J., Richtera L., Nejdl L., Xhaxhiu K., Vitek P., Rutkay-Nedecky B., Hynek D., Kopel P., Adam V., Kizek R. (2016). Improved Electrochemical Detection of Zinc Ions Using Electrode Modified with Electrochemically Reduced Graphene Oxide. Materials.

[50] Brett C.M.A. (2001). Electrochemical Sensors for Environmental Monitoring. Strategy and Examples. Pure Appl. Chem..

[51] Lin M., Hu X., Pan D., Han H. (2018). Determination of Iron in Seawater: From the Laboratory to in Situ Measurements. Talanta.

[52] Lin M., Pan D., Zhu Y., Hu X., Han H., Wang C.C. (2016). Dual-Nanomaterial Based Electrode for Voltammetric Stripping of Trace Fe(II) in Coastal Waters. Talanta.

[53] Kubyshkin A.P. (1994). Photothermal Measurement of Bulk and Surface Absorption of Transparent Infrared Optical Elements. Opt. Eng..

[54] Commandré M., Roche P. (1995). Characterization of Absorption by Photothermal Deflection. Thin Film. Opt. Syst..

[55] Soumya S., Arun Kumar R., Raj V., Swapna M.S., Sankararaman S. (2021). Thermal Diffusivity of Molybdenum Oxide Nanowire Film: A Photothermal Beam Deflection Study. Opt. Laser Technol..

[56] Sell J. (2012). Photothermal Investigations of Solids and Fluids.

[57] Li B., Welsch E. (1999). Configuration Optimization and Sensitivity Comparison among Thermal Lens, Photothermal Deflection, and Interference Detection Techniques. Proceedings of the Laser-Induced Damage in Optical Materials.

[58] Zhang X., Li B. (2018). Configuration Optimization of Photothermal Deflection for Measurement Sensitivity Enhancement. Rev. Sci. Instrum..

[59] Jackson W.B., Amer N.M., Boccara A.C., Fournier D. (1981). Photothermal Deflection Spectroscopy and Detection. Appl. Opt..

[60] Budasheva H., Kravos A., Korte D., Bratkič A., Gao Y., Franko M. (2019). Determination of Dissolved Iron Redox Species in Freshwater Sediment Using DGT Technique Coupled to BDS. Acta Chim. Slov..

[61] Warnken K.W., Zhang H., Davison W. (2004). Performance Characteristics of Suspended Particulate Reagent-Iminodiacetate as a Binding Agent for Diffusive Gradients in Thin Films. Anal. Chim. Acta.

[62] Ding S., Xu D., Sun Q., Yin H., Zhang C. (2010). Measurement of Dissolved Reactive Phosphorus Using the Diffusive Gradients in Thin Films Technique with a High-Capacity Binding Phase. Environ. Sci. Technol..

[63] Buckley J.A. (1985). Preparation of Chelex-100 Resin for Batch Treatment of Sewage and River Water at Ambient PH and Alkalinity. Anal. Chem..

[64] Lin T.-S., Nriagu J.O. (1999). Thallium Speciation in River Waters with Chelex-100 Resin. Anal. Chim. Acta.

[65] Nomngongo P.N., Catherine Ngila J., Msagati T.A.M., Moodley B. (2013). Preconcentration of Trace Multi-Elements in Water Samples Using Dowex 50W-X8 and Chelex-100 Resins Prior to Their Determination Using Inductively Coupled Plasma Atomic Emission Spectrometry (ICP-OES). Phys. Chem. Earth Parts A/B/C.

[66] Leermakers M., Gao Y., Gabelle C., Lojen S., Ouddane B., Wartel M., Baeyens W. (2005). Determination of High Resolution Pore Water Profiles of Trace Metals in Sediments of the Rupel River (Belgium) Using Det (Diffusive Equilibrium in Thin Films) and DGT (Diffusive Gradients in Thin Films) Techniques. Water. Air. Soil Pollut..

[67] Korte D., Franko M. (2015). Application of Complex Geometrical Optics to Determination of Thermal, Transport, and Optical Parameters of Thin Films by the Photothermal Beam Deflection Technique. J. Opt. Soc. Am. A.

[68] Korte D., Carraro G., Fresno F., Franko M. (2014). Thermal Properties of Surface-Modified α-and ε-Fe 2 O 3 Photocatalysts Determined by Beam Deflection Spectroscopy. Int. J. Thermophys..

[69] Swapna M.N.S., Korte D., Sankararaman S.I. (2022). Unveiling the Role of the Beam Shape in Photothermal Beam Deflection Measurements: A 1D and 2D Complex Geometrical Optics Model Approach. Photonics.

[70] Pai S.-C., Whung P.-Y., Lai R.-L. (1988). Pre-Concentration Efficiency of Chelex-100 Resin for Heavy Metals in Seawater: Part 1. Effects of PH and Salts on the Distribution Ratios of Heavy Metals. Anal. Chim. Acta.

[71] Franko M., Goljat L., Liu M., Budasheva H., Žorž Furlan M., Korte D. (2023). Recent Progress and Applications of Thermal Lens Spectrometry and Photothermal Beam Deflection Techniques in Environmental Sensing. Sensors.

[72] Caputi L., Carradec Q., Eveillard D., Kirilovsky A., Pelletier E., Pierella Karlusich J.J., Rocha Jimenez Vieira F., Villar E., Chaffron S., Malviya S. (2019). Community-Level Responses to Iron Availability in Open Ocean Plankton Ecosystems. Global Biogeochem. Cycles.

[73] Birchill A.J., Hartner N.T., Kunde K., Siemering B., Daniels C., González-Santana D., Milne A., Ussher S.J., Worsfold P.J., Leopold K. (2019). The Eastern Extent of Seasonal Iron Limitation in the High Latitude North Atlantic Ocean. Sci. Rep..

[74] Geng H., Wang F., Yan C., Ma S., Zhang Y., Qin Q., Tian Z., Liu R., Chen H., Zhou B. (2022). Rhizosphere Microbial Community Composition and Survival Strategies in Oligotrophic and Metal(Loid) Contaminated Iron Tailings Areas. J. Hazard. Mater..

[75] Wei H., Xu L., Su J., Liu S., Zhou Z., Li X. (2024). Simultaneous Removal of Nitrogen, Phosphorus, and Organic Matter from Oligotrophic Water in a System Containing Biochar and Construction Waste Iron: Performances and Biotic Community Analysis. Environ. Res..

[76] Moosavi S.M., Ghassabian S. (2018). Linearity of Calibration Curves for Analytical Methods: A Review of Criteria for Assessment of Method Reliability.

[77] García-Miranda Ferrari A., Carrington P., Rowley-Neale S.J., Banks C.E. (2020). Recent Advances in Portable Heavy Metal Electrochemical Sensing Platforms. Environ. Sci. Water Res. Technol..

[78] Zhou C., van de Velde S., Baeyens W., Gao Y. (2018). Comparison of Chelex Based Resins in Diffusive Gradients in Thin-Film for High Resolution Assessment of Metals. Talanta.

[79] Greenland S., Senn S.J., Rothman K.J., Carlin J.B., Poole C., Goodman S.N., Altman D.G. (2016). Statistical Tests, P Values, Confidence Intervals, and Power: A Guide to Misinterpretations. Eur. J. Epidemiol..

[80] Sochaczewski L., Tych W., Davison B., Zhang H. (2007). 2D DGT Induced Fluxes in Sediments and Soils (2D DIFS). Environ. Model. Softw..

[81] Sochaczewski Ł., Davison W., Zhang H., Tych W. (2009). Understanding Small-Scale Features in DGT Measurements in Sediments. Environ. Chem..

[82] Zhang H., Davison W., Mortimer R.J.G., Krom M.D., Hayes P.J., Davies I.M. (2002). Localised Remobilization of Metals in a Marine Sediment. Sci. Total Environ..

[83] Zhou Y., Wang H., Zhang Y., Cai Y., Yin H., Yang Z., Li Q., Yuan H. (2021). Availability and Diffusion Kinetic Process of Phosphorus in the Water–Sediment Interface Assessed by the High-Resolution DGT Technique. J. Soils Sediments.

[84] Morford J., Kalnejais L., Martin W., François R., Karle I.-M. (2003). Sampling Marine Pore Waters for Mn, Fe, U, Re and Mo: Modifications on Diffusional Equilibration Thin Film Gel Probes. J. Exp. Mar. Bio. Ecol..

[85] Bennett W.W., Teasdale P.R., Welsh D.T., Panther J.G., Stewart R.R., Price H.L., Jolley D.F. (2011). Inorganic Arsenic and Iron (II) Distributions in Sediment Porewaters Investigated by a Combined DGT–Colourimetric DET Technique. Environ. Chem..

[86] Nagakura T., Schubert F., Wagner D., Kallmeyer J. (2022). Biological Sulfate Reduction in Deep Subseafloor Sediment of Guaymas Basin. Front. Microbiol..

[87] Zhou T., Wang Z., Hu Q., Liu L., Luo K. (2016). Effects of Fe^2+^ and Fe^3+^ on Algal Proliferation in a Natural Mixed Algal Colony in Algae-Rich Raw Water in Southern China. J. Residuals Sci. Technol..

[88] Lueder U., Jørgensen B.B., Maisch M., Schmidt C., Kappler A. (2022). Influence of Fe(III) Source, Light Quality, Photon Flux and Presence of Oxygen on Photoreduction of Fe(III)-Organic Complexes—Implications for Light-Influenced Coastal Freshwater and Marine Sediments. Sci. Total Environ..

[89] Daniel Chukwuemeka O., Innocent Onyebuchi E. (2021). REDOX PROCESSES IN REGOLITH AQUIFERS AT ENUGU. Int. J. Earth Environ. Sci..

[90] Korte D., Tomsič G., Bratkič A., Franko M., Budasheva H. (2019). Determination of Iron in Environmental Water Samples by FIA-TLS. Acta Chim. Slov..

